# Inference of mechanical states of intestinal motor activity using hidden Markov models

**DOI:** 10.1186/1472-6793-13-14

**Published:** 2013-12-11

**Authors:** Lukasz Wiklendt, Marcello Costa, Phil G Dinning

**Affiliations:** 1 Discipline of Human Physiology, Flinders Medical Science and Technology, Flinders University, Adelaide, Australia; 2 Departments of Gastroenterology and Surgery, Flinders Medical Centre, Adelaide, Australia

**Keywords:** Manometry, Peristalsis, Time-series analysis, Muscle mechanics

## Abstract

**Background:**

Contractions and relaxations of the muscle layers within the digestive tract alter the external diameter and the internal pressures. These changes in diameter and pressure move digesting food and waste products. Defining these complex relationships is a fundamental step for neurogastroenterologists to be able define normal and abnormal gut motility.

**Results:**

Utilising an in vitro technique that allows for the simultaneous recording of intraluminal pressure (manometry) and gut diameter (video) in an isolated section of rabbit colon, we developed a technique to help define the mechanical states of the muscle at any point in space and time during actual peristaltic movements. This was achieved by directly relating the changes in pressure to the changes in diameter along the length of the gut studied. For each individual measure of pressure or diameter, 3 dynamic state components were identified; increasing or decreasing changes or a stable period. Two additional static state components, fully contracted and fully distended, were defined for the diameter. Then qualitative mechanical states of the muscle activity were defined as combinations of these state components. A hidden Markov model was used to correlate adjacent-in-time samples, and the Viterbi algorithm was used to infer the most likely sequence of mechanical states based on the observed data. From this a spatiotemporal map of the mechanical states was produced, showing the regions of active contractions, active relaxations, or passive states along the length of the gut throughout the entire recording period.

**Conclusions:**

The identification of mechanical muscles states based on gut diameter and intraluminal pressure was possible by modelling muscle activation with a hidden Markov model.

## Background

**Figure 1:**
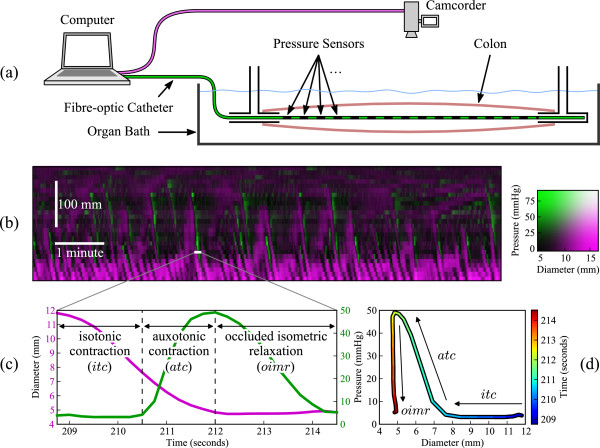
**An overview of data acquisition, and how mechanical muscle states relate to the data.(a)** A schematic of the experimental setup. A section of rabbit colon is kept alive in an organ bath and simultaneous intraluminal pressure (fibre-optic catheter, sensors spaced at 10 mm) and diameter (camcorder) are recorded during peristaltic movements. **(b)** from these recordings a diameter (magenta) and pressure (green) spatiotemporal map is created. **(c)** at any location within the diameter-pressure map we can plot the values of pressure (green) and diameter (magenta). In this example the values are taken from the 5s white line on the diameter-pressure map. **(d)** Plotting the relation between pressure and diameter in **(c)** is then displayed as an orbit plot. From the orbit plot the mechanical states of the muscle can be determined. In the example shown here 3 different mechanical states can been observed during the selected 5s period. The first mechanical state (blue–aqua section of the line) shows a decrease in diameter with no change in pressure (isotonic contraction; *itc*). In the next mechanical state (aqua–yellow) the pressure begins to increase with a corresponding decease in diameter (auxotonic contraction; *atc*). In the final mechanical state (yellow–red) the pressure drops with no further change in diameter (occluded isometric relaxation; *oimr*).

The gastrointestinal tract extends from the mouth to the anus. Swallowed food travels along the length of this tube at an appropriate speed to allow for the breakdown and absorption of nutrients. The movement of content results from a complex series of muscular contractions and relaxations. These movements of the gut wall alter the pressure profiles within the gut [1] which in-turn cause the digesting contents to move in an oral or anal direction. Abnormalities in these motor patterns are associated with several prevalent and unpleasant disorders that cost health care systems billions of dollars per year [2]. The ability to define the relationship that exist between wall motion, intraluminal pressure and the flow of content is a fundamental step in understanding how altered motor patterns effect the transport of luminal content. However, accurately defining these relationships in the human gut *in vivo* is problematic; ethical constraints prevent detailed examination of real-time movement of the gut wall. To overcome this problem we developed an *in vitro* animal preparation that allowed us to record simultaneously, both intraluminal pressure (high-resolution manometry) and gut diameter (video) in real time, across varying length (15–90 cm) of intestine [3] (Figure 1a,b).

In the next stage of this research we published a theory based paper in which we developed a strategy based on simple principle of biomechanics to deduce the mechanical state of the muscle (active contraction or relaxation, passive dilation, periods of quiescence, etc.) by calculating the relation between pressure and diameter at every point along the gut segment, and establishing where and when the muscle is actively contracting or relaxing [4] (Figure 1c,d). In that paper, space prevented a detailed description of the mathematical model involved in developing this process. In this work we address the details of the strategy that enabled us to confidently identify, for the first time, the mechanical states of the muscle during peristaltic contractions, and plot them as a functional spatiotemporal map.

A major factor in our work involved the use of hidden Markov models (HMM). The HMM have found extensive use in a variety of fields, stemming from their seminal application in speech recognition in the 1970s [5]. In gastroenterology, HMMs have been used to classify the location of a video capsule in the gastrointestinal tract [6]. In their application to hand written recognition, HMMs were utilized by observing lines and curves drawn on a 2D plane and inferring the most probable characters they represent [7]. In developing our intestinal model of mechanical states, one of the major challenges we faced was how to identify the beginning and the end of the periods of the various different states of the muscle. However, analogous to hand written recognition, our diameter-pressure plots can be observed as lines in the 2D plane. Therefore the HMM was adapted to infer the most probable muscles states at any given point in time along the length of the studies gut segment. A description of the steps involved in this process are detailed here.

### Identification of muscle states

**Figure 2:**
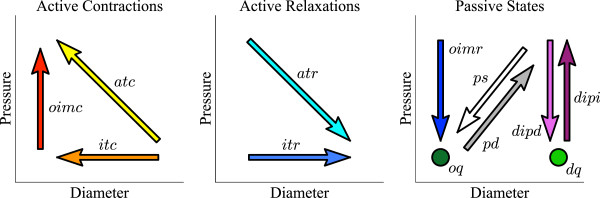
**Conceptual directions of observed data and their relation to mechanical muscle states.** The left and middle graphs correspond to states where myogenic, or neurogenic excitatory or inhibitory mechanisms are active. The graph on the right corresponds to passive states where no muscle activation is required to exhibit that behaviour.

The circular muscle of the gut is arranged as continuous rings of smooth muscle forming the inner muscle layer of the tubular structure. The circular muscle layer is thicker than the external longitudinal muscle layer and thus is mechanically more powerful. When it contracts, propulsion of the luminal content can occur. This prolusion is made more efficient if the adjacent regions of the gut (proximal or distal) concurrently relax, thus effectively allowing the bolus to move into a region of lower pressure [8]. These regions of contraction and relaxation can be detailed by simultaneously recording the diameter of the gut and the intraluminal pressure (Figure 1a). From the resultant spatiotemporal map of diameter and pressure (Figure 1b) we can plot, at any point in time, pressure and diameter curves (Figure 1c). From these we can then begin to express the mechanical relation between pressure and diameter as a pressure-diameter orbit plot (Figure 1d). In publishing the theory behind this concept [4] we also established that twelve distinct mechanical muscle states can be predicted (Figure 2). The mechanical states are summarized below; **Occluded isometric contraction** (*oimc*): active; occurs with an increase in pressure, with no change in diameter, when the gut is at its minimum diameter. **Occluded isometric relaxation** (*oimr*): passive; occurs with a decrease in pressure and no change in diameter, when the gut is at its minimum diameter. **Distended isometric pressure increase** (*dipi*): passive; occurs with an increase in pressure, no change in diameter, when the gut is at its maximum diameter. **Distended isometric pressure decrease** (*dipd*): passive; occurs with a decrease in pressure, no change in diameter, and a maximum diameter. **Isotonic contraction** (*itc*): active; occurs with a decrease in diameter and no change in pressure. **Isotonic relaxation** (*itr*): active; occurs with an increase in diameter and no change in pressure. **Auxotonic contraction** (*atc*): active: occurs with a decrease in diameter and an increase in pressure. **Auxotonic relaxation** (*atr*): active: occurs with an increase in diameter and a decrease in pressure. **Passive shortening** (*ps*): passive; occurs with a decrease in diameter and pressure. **Passive dilation** (*pd*): passive; occurs with an increase in diameter and pressure. **Occluded quiescence** (*oq*): passive; occurs with no change in diameter nor pressure, when the gut is at its minimum diameter. **Distended quiescence** (*dq*): passive; occurs with no change in diameter nor pressure, when the gut is at a non-minimum diameter.

Having established that 12 mechanical states exist we then need to develop a means to determine when and where each state existed along the gut in an automated fashion. In addition we had to detect both the changes in the mechanical states (transitions of direction of the linear segments of the orbits) and the permanence in that state (the beginning and end of the linear segment).

## Methods

The intraluminal pressures were recorded by a manometry catheter with pressure sensors spaced at 10 mm intervals and each of these was considered independent for modelling purposes. At each sensor location, diameter and pressure values were recorded continuously.

### Data acquisition

A detailed description of the techniques used to collate the data has been published elsewhere [4]. Here we will provide a brief overview.

Rabbits were euthanized humanely by intravenous injection of pentobarbitone sodium (0.5 ml kg^−1^) in accordance with approval by the Animal Welfare Committee of Flinders University.

The gut diameter and internal pressure were recorded using two different methods, at different sampling rates, spacial resolutions, and offsets in time and space. Resampling and spatiotemporal alignment was required to coincide values of pressure and diameter for use as a single observation vector for each node in the Markov chain. Each spatial location was assigned its own Markov chain.

A spatiotemporal map of colonic diameter was based on techniques developed in our lab [9]. Briefly the spatiotemporal maps were obtained by recording a top-down video of the colon suspended in a bath of Krebs solution, such that the length of the colon appeared horizontally in the video, and the number of pixels that the colon spans for each vertical pixel line in each frame was counted. Using a reference ruler visible in the recorded video, the colonic diameter in millimetres was obtained.

Pressures were recorded by 10 mm-spaced sensors at 10 Hz with a catheter inserted into the colon. Baseline drift was removed with iterated Gaussian minima smoothing [10].

Different spatiotemporal resolutions and offsets required resampling and alignment. A diameter map and a pressure map were combined by creating a grid of coordinates using the coarsest resolution in time and space from either map aligned with an adjustable spatiotemporal offset, and then binning the original maps into the grid. This resulted in a spatial and time resolution of 10 mm and 0.25 s respectively.

The alignment resulted in a single set of spatiotemporal coordinates that map to coincident diameter and pressure values, allowing diameters and pressures to be quickly compared for analysis without further interpolation. The temporal offset was determined by aligning events that were synchronously recorded by video as flashes from a light bulb (for diameter data) and embedded into the pressure recording as meta-data. The spatial offset was manually obtained by observing overlapping images of the two maps, while adjusting the offset to a value best representative of correct alignment that correlates with the video.

The entire data set consisted of 6 rabbits recorded with approximately 20 sensors over periods of 10 to 20 minutes each, resulting in just over 420,000 samples (almost 30 hours); orbits were created for each sensor.

### Hidden Markov model

Given a sequence of observations of diameter and pressure at a single location in the colon, our objective was to infer the most-likely sequence of mechanical muscle states that could have resulted in those observations. Each mechanical muscle state was directly represented as a hidden state in the hidden Markov model. In our application, the dependence of a state on the previous state in the Markov chain was required as an implicit smoothing technique.

#### Observations

For each sensor location there are two values observed, the diameter *d* and pressure *p*, sampled at a frequency of 4 Hz. Sample *i* of the diameter and pressure is given by *d*_*i*_ and *p*_*i*_, where *i*∈{1…*N*}. The time derivatives of those values are denoted with an overdot and estimated using central differences as

(1) $$\begin{array}{ll} {\dot{d}}_{i} & \approx \frac{d_{i+1}-d_{i-1}}{2\Delta t}\; \end{array}$$

(2) $$\begin{array}{ll} {\dot{d}}_{1} & \approx \frac{d_{2}-d_{1}}{\Delta t}\; \end{array}$$

(3) $$\begin{array}{ll} {\dot{d}}_{N} & \approx \frac{d_{N}-d_{N-1}}{\Delta t}\; \end{array}$$

where *Δt*=0.25*s* for the 4 Hz sampling, and $\dot{p}$ defined in the same manner as $\dot{d}$.

Observation *i* is defined as the vector

(4) $$o_{i}=\left(d_{i},{\dot{d}}_{i},{\dot{p}}_{i}\right)$$

Any general sequence (*x*_*a*_, *x*_*a*+1_, …, *x*_*b*−1_, *x*_*b*_) will be written as *x*_*a*:*b*_ for the sake of brevity.

#### States

We segment a sensor’s recording and classify each observation into one of twelve discrete mechanical states of the set

$$\begin{array}{lll} \;S=\{ & \text{oimc},\text{oimr},\text{dipi},\text{dipd},\text{itc},\text{itr},\; & \;\\ & \text{atc},\text{atr},\text{ps},\text{pd},\text{oq},\text{dq}\}\; & \; \end{array}$$

The conceptual directions and positions of subsequences of observations on a diameter-pressure plot representing examples of each of the twelves states is depicted in Figure 2 and described in Figure 1a. The state-observation model is based on developing a quantitative model for classifying the dynamics depicted in that figure.

#### State-observation model

The state-observation model defines the conditional probabilities of observations and states. The goal is to arrive at a formula describing the distribution of possible observations, under the assumption that they were produced while the muscle was in any given mechanical state. The sample subscript *i* is omitted for brevity in this section, since the state-observation model is independent of the sample number.

Each state in *S* is composed of 3 state components, where each component *c*_*o*_ corresponds to an element *o* of the observation **o**, and non corresponding components and elements are considered independent. The components *quiet*, *pos*, and *neg* correspond to the $\dot{d}$ and $\dot{p}$ velocity elements. The *quiet* component corresponds to a value of the velocity element close to 0, and *pos* and *neg* represent the positive and negative values. The components *occ*, *dis*, and *any* correspond to the diameter *d*, where *occ* represents an occluded diameter, *dis* a distended diameter, and *any* if the diameter is irrelevent. For example, the state *dipd* is composed of *dis*, ${\text{quiet}}_{\dot{d}}$, and ${\text{neg}}_{\dot{p}}$, where the subscripts disambiguate the corresponding velocity elements.

The states in *S* can be considered a vector of state components $\left(c_{d},c_{\dot{d}},c_{\dot{p}}\right)$, where

(5) $$\begin{array}{ll} c_{d} & \in \{\text{any},\text{dis},\text{occ}\}\; \end{array}$$

(6) $$\begin{array}{ll} c_{\dot{d}} & \in \{{\text{quiet}}_{\dot{d}},{\text{pos}}_{\dot{d}},{\text{neg}}_{\dot{d}}\}\; \end{array}$$

(7) $$\begin{array}{ll} c_{\dot{p}} & \in \{{\text{quiet}}_{\dot{p}},{\text{pos}}_{\dot{p}},{\text{neg}}_{\dot{p}}\}\; \end{array}$$

Given a velocity observation $v\in \{\dot{d},\dot{p}\}$, the probability of the corresponding state component being *quiet* is modelled on the basis of a normal distribution located at 0 with width free parameters $\sigma_{\dot{d}}$ and $\sigma_{\dot{p}}$. The normal distribution is the most common way to model noise and errors, justified by the central limit theorem. Since very low speeds are considered quiescent, slow tonic muscle activity will be absorbed into passive states, with only phasic contractions exposed as active states. The probability of the state component being *pos* or *neg* is modelled on the basis of normal cumulative distribution functions with the same parameters. Probability mass functions are normalised to ensure

(8) f(quiet|v)+f(pos|v)+f(neg|v)=1

**Figure 3:**
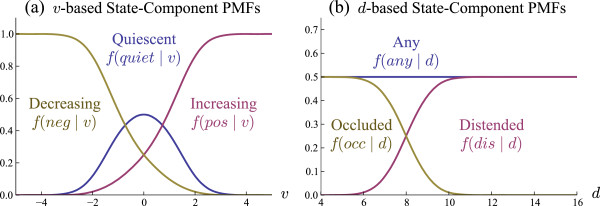
**An example of the PMFs defined by (10)–(15).** Given an observation of $v\in \{\dot{d},\dot{p}\}$ or *d*, the functions model the probability that the observation was generated from the state pertaining to each function. **(a)** shows examples of velocity-based component PMFs (10)–(12) with parameter *σ*_*v*_ = 1. **(b)** shows examples of diameter-based component PMFs (13)–(15) with parameters *σ*_*d*_ = 1, *μ*_*d*_ = 8, and min_*d*_ = 0.

See (10)–(12) for the formulae, and Figure 3 (a) for a visual example.

Given an observation of diameter *d*, the probability of the corresponding state component being *occ* or *dis* is modelled on the basis of a normal cumulative distribution function and its complement, located at *μ*_*d*_+min_*d*_ with width *σ*_*d*_, where min_*d*_ is the value of the minimum recorded diameter. The value *d*−min_*d*_ is referred to as “dilation”, and *μ*_*d*_ represents the amount of dilation that separates occluded and distended states. The probability of the state being *any* given *d* is a constant. The probability mass functions are normalised to ensure

(9) f(occ|d)+f(dis|d)+f(any|d)=1

See (13)–(15) for the formulae, and Figure 3 (b) for a visual example.

The probability mass functions (PMFs) *f*(*c*_*o*_|*o*) are given by

(10) $$\begin{array}{ll} f(\text{quiet}|v) & =Z_{v}\;\exp\left(-\frac{v^{2}}{2\overset{2}{\underset{v}{\sigma }}}\right)\; \end{array}$$

(11) $$\begin{array}{ll} f(\text{pos}|v) & =Z_{v}\;\Phi \left(\frac{v}{\sigma_{v}}\right)\; \end{array}$$

(12) $$\begin{array}{ll} f(\text{neg}|v) & =Z_{v}\;\Phi \left(\frac{-v}{\sigma_{v}}\right)\; \end{array}$$

(13) $$\begin{array}{lll} Z_{v} & ={\left(\exp\left(-\frac{v^{2}}{2\overset{2}{\underset{v}{\sigma }}}\right)+1\right)}^{-1}\; & \;\\ f(\text{any}|d) & =Z_{d}\;1\; \end{array}$$

(14) $$\begin{array}{ll} f(\text{dis}|d) & =Z_{d}\;\Phi \left(\frac{d-{\text{min}}_{d}-\mu_{d}}{\sigma_{d}}\right)\; \end{array}$$

(15) $$\begin{array}{ll} f(\text{occ}|d) & =Z_{d}\;\Phi \left(\frac{\mu_{d}-d+{\text{min}}_{d}}{\sigma_{d}}\right)\; \end{array}$$

$$\begin{array}{lll} Z_{d} & =\frac{1}{2}\; & \; \end{array}$$

where *Φ* is the cumulative standard normal distribution function, *v* is an alias for either $\dot{d}$ or $\dot{p}$, and *Z*_*v*_ and *Z*_*d*_ are normalisation constants ensuring the probabilities sum to 1 for any given observation and all corresponding states.

The probability density function (PDF) of the model emitting a particular observation element $o\in \{d,\dot{d},\dot{p}\}$ given a corresponding state component *c*_*o*_, is known as the *emission PDF*, which can be obtained from Bayes’ theorem

(16) $$f(o|c_{o})=\frac{f(c_{o}|o)f(o)}{f(c_{o})}$$

The emission PDF is used to determine the distribution of observations that can be made assuming an underlying muscle mechanical state. Since only the relative sizes of emission PDFs are required for calculating the optimal state sequence (rather than the actual probability values themselves), the observation prior density *f*(*o*) can be removed which is independent of state and so does not change which state is most likely to have produced a given observation. In a similar way, if we approximate the state component prior distribution *f*(*c*_*o*_) with a constant, then it can also be removed resulting in the following practical approximation

(17) $$f(o|c_{o})\approx f(c_{o}|o)$$

Note that under this approximation, *f*(*o*|*c*_*o*_) no longer represents a PDF, that is, $\int f(o|c_{o})\text{do}\neq 1$.

This approximation isn’t necessary for performance, since finding the priors numerically is straight forward with Gaussian kernel density estimation of *f*(*o*) and the marginalization $f(c_{o})=\int f(c_{o}|o)f(o)\text{do}$, which resulted in priors of *f*(*c*_*o*_) = 0.33 ± 0.09 for our data. Rather it is our desire to keep the emission PDFs free of any other data-based attributes besides the few parameters that were chosen by hand. This allows for the consistent interpretation of results by having a homogeneous muscle mechanics model which is comparable among different preparations and recordings.

Assuming independence of the variables *d*, $\dot{d}$, and $\dot{p}$, the joint emission PDF can be factorized into

(18) $$\begin{array}{lll} f(o|s) & =f(d,\dot{d},\dot{p}|s)\; & \;\\ & =f(d|c_{d})\;f(\dot{d}|c_{\dot{d}})\;f(\dot{p}|c_{\dot{p}})\; \end{array}$$

where the model of the state $s=\left(c_{d},c_{\dot{d}},c_{\dot{p}}\right)$ subtends the following specific factorisation associations

$$\begin{array}{ccccc} \text{oimc} & = & (\text{occ}, & \text{quiet}, & \text{pos})\\ \text{oimr} & = & (\text{occ}, & \text{quiet}, & \text{neg})\\ \text{dipi} & = & (\text{dis}, & \text{quiet}, & \text{pos})\\ \text{dipd} & = & (\text{dis}, & \text{quiet}, & \text{neg})\\ \text{itc} & = & (\text{any}, & \text{neg}, & \text{quiet})\\ \text{itr} & = & (\text{any}, & \text{pos}, & \text{quiet})\\ \text{atc} & = & (\text{any}, & \text{neg}, & \text{pos})\\ \text{atr} & = & (\text{any}, & \text{pos}, & \text{neg})\\ \text{ps} & = & (\text{any}, & \text{neg}, & \text{neg})\\ \text{pd} & = & (\text{any}, & \text{pos}, & \text{pos})\\ \text{oq} & = & (\text{occ}, & \text{quiet}, & \text{quiet})\\ \text{dq} & = & (\text{dis}, & \text{quiet}, & \text{quiet}) \end{array}$$

with implicit substitutions of (10)–(12) and (13)–(15) to be made in (18) due the approximation (17). The independence assumption is a model simplification rather than an observed independence in the data, and allows for the factorization of the joint emission PDF into component emission PDFs. Making such an independence assumption, where none exists in reality, is common practice in machine learning for simplifying models and algorithms used to make inference. In practice, inference algorithms are often robust despite such assumptions, and we observe that inferences obtained by our technique remain consistent with expected states.

#### Optimal state sequence

Given an observation sequence **o**_1:*N*_, our goal is to find the optimal sequence of states *s*_1:*N*_ that explains the observations. The joint PDF of the observation and state sequences is given by a hidden Markov model [5], which subsumes the following factorisation

(19) $$\begin{array}{ll} f(o_{1:N},s_{1:N}) & =f(o_{1}|s_{1})f(s_{1})\overset{N}{\underset{i=2}{\prod }}f(o_{i}|s_{i})f(s_{i}|s_{i-1})\; \end{array}$$

To keep the number of free parameters small, we choose the state transition probabilities to be independent of state for non self-transitions, given by

(20) $$f(s_{i}|s_{i-1})=\left\{\begin{array}{ll} \gamma , & \;\text{if}\;\;s_{i}=s_{i-1}\\ \frac{1-\gamma }{|S|-1}, & \;\text{otherwise} \end{array}\right)$$

where *γ* ∈ (0, 1) is a free parameter that represents a penalty (when $\gamma >\frac{1}{\left|S\right|}$) for changing states between samples. Such a penalty allows the inferred state sequence to be desensitised to noisy observations, resulting in practical smoothing of the sequence. It is important to include this implicit smoothing so that linear segments of orbits containing undulations are not subdivided, which may result in incorrect state inferences based on arbitrary angles of small subsegments of the undulated linear segment. A value of $\gamma =\frac{1}{\left|S\right|}$ would result in modelling the states in adjacent samples as independent, effectively eliminating the links in the Markov chain.

**Figure 4:**
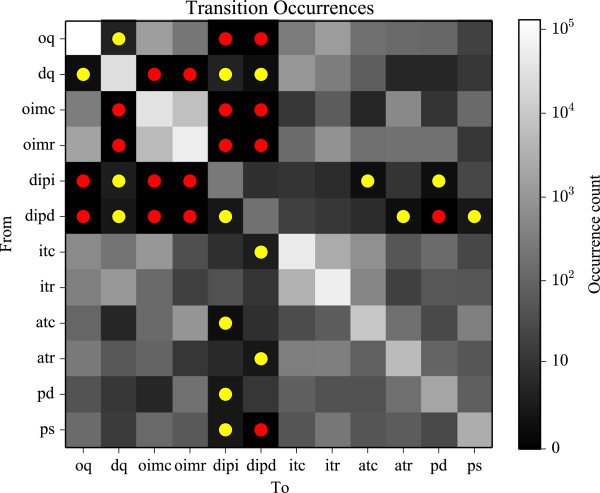
**A matrix of state transition occurrence counts.** The vertical and horizontal axes represent each of the 12 states, such that a transition moving from one state (vertical axis) to another state (horizontal axis) is represented as a square shaded from black (0 occurrences of that transition) to white (many occurrences of that transition). Transitions that never occurred are marked with a red circle, and transitions that occurred only very rarely (between 1 and 4 times for the entire data set) are marked with a yellow circle.

Due to the difficulty in quantifying and translating physically realistic dynamics into transition probabilities under our model, state-dependent transition probabilities were not used. However, by plotting all of the transition occurrences from our complete data set (over 420,000 samples) we have been able to demonstrate that qualitatively unlikely transitions were either not observed or rarely observed in the inference results (Figure 4). For example we did not have a single example of a mechanical state moving from occluded quiescence to a distended isometric pressure increase. Thus we are confident that state-independent transition probabilities are sufficient.

The probability of the initial state is modelled with the discrete uniform distribution

(21) $$f(s_{1})=\frac{1}{\left|S\right|}$$

The solution to the most likely sequence of states for a given sequence of observations is obtained by performing the following maximisation

(22) $$\arg\;\underset{s_{1:N}}{\text{max}}f(s_{1:N}|o_{1:N})$$

where *f*(*s*_1:*N*_ | **o**_1:*N*_) is proportional over *s*_1:*N*_ to (19), and the solution can be found by applying the Viterbi algorithm. The Viterbi algorithm works by calculating the most likely sequence of states based on a sequence of discrete observations. To handle continuous observations we used the value of the emission PDF at each observation as a substitute for the probability of making that observation.

#### Parameters

Through an iterative trial-and-error approach we manually selected and tuned parameter values, checking selected areas of data where we could verify whether the inference resulted in states consistent with existing domain knowledge of the expected muscular activity. For example, when the gut contracts and squeezes the catheter we expect the pressure to rise. For this approach, data was drawn from a total of 6 rabbits, each with 10 to 20 minutes of recording. The values for parameters are given in the middle column of Table 1.

**Table 1:** Parameter values used in inference and analysis

**Parameter**	**Values for inference**	**Analysis range**
*μ*_*d*_	2.5 mm	0–7.5 mm
*σ*_*d*_	0.4 mm	0–1.2 mm
$\sigma_{\dot{d}}$	0.4 mm/s	0–1.2 mm/s
$\sigma_{\dot{p}}$	3 mmHg/s	0–9 mmHg/s
*γ*	0.5	0.255–0.99

To test the classification sensitivity of different parameter values to the ones selected manually, parameters were varied one at a time through a range of values (right column in Table 1). One *reference* classification, considered as ground-truth, was performed with the manually selected parameter values (middle column in Table 1). The reference classification was compared to many *comparison* classifications with the varied parameter values. A ratio *ε* of the number of incorrect active-state classifications to the total number of states which were active in both reference and comparison classification over the entire spatiotemporal map was given by the equation

(23) $$\begin{array}{ll} \varepsilon & =\frac{1}{\underset{j,i}{\sum }\alpha_{\text{ji}}}\underset{j,i}{\sum }\alpha_{\text{ji}}\left[{ŝ}_{\text{ji}}\neq {ŝ}_{\text{ji}}^{\prime }\right]\; \end{array}$$

(24) $$\begin{array}{ll} \alpha_{\text{ji}} & =1_{\left\{\text{ac},\text{ar}\right\}}({ŝ}_{\text{ji}})\;1_{\left\{\text{ac},\text{ar}\right\}}({ŝ}_{\text{ji}}^{\prime })\; \end{array}$$

where the square brackets denote Iverson brackets^a^, **1** is the indicator function^b^, *i* and *j* denote the sample and sensor number (horizontal and vertical position in the spatiotemporal map) respectively. A simplified state ${ŝ}_{j,i}\in \{\text{ac},\text{ar},\text{ps}\}$ is one of: active contraction *ac* = {*oimr*, *itc*, *act*}, active relaxation *ar* = {*itr*, *atr*}, or passive states *ps* = {*oimr*, *dipi*, *dipd*, *ps*, *pd*, *oq*, *dq*}. A simplified state in the comparison classification is denoted with a prime ${ŝ}_{j,i}^{\prime }$.

**Figure 5:**
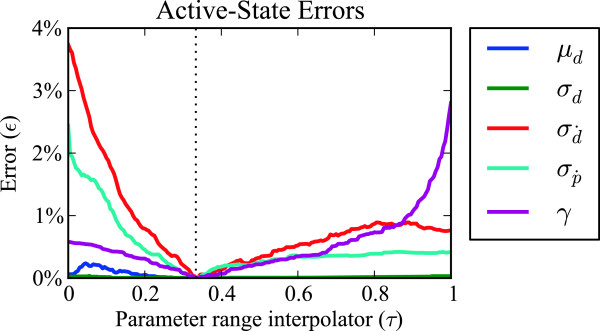
**Comparison of the reference parameter values to values varying within the ranges shown in Table **1**.** Small errors *ε* based on equation (23) show that the model is robust to variations in parameter values compared to those selected manually. Parameter ranges were mapped to [0,1] in the figure so that the manually selected values all coincided at the same location $\tau =\frac{1}{3}$ for a clearer visual comparison.

Results on the error *ε* as parameter values vary (Figure 5) shows that the model is adequately robust to variations in the parameters, as the errors are qualitatively considered small. The test was performed on a 10-minute recording with 26 sensors of a single isolated rabbit colon which exhibited typical activity (for a combined 62400 samples in total).

## Results and discussion

**Figure 6:**
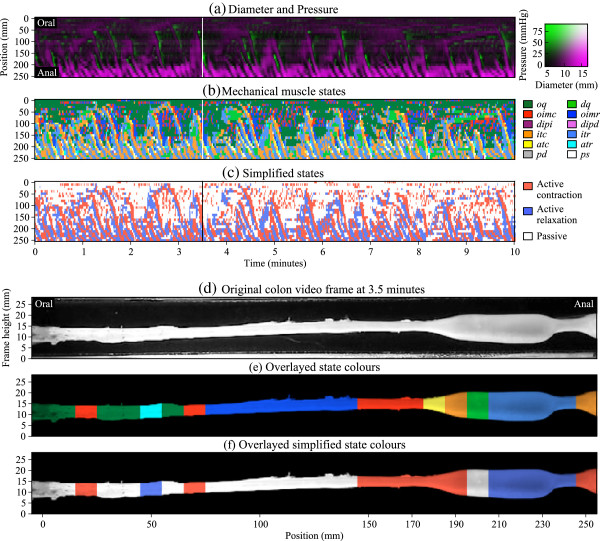
**Results for a 10 minute recording of an *in vitro *rabbit distal colon. (a)** a composite Diameter/Pressure Map (DPMap). In **(b)** each of the 12 possible mechanical states are mapped to different colors. The map portrays the periods of quiescence either when the gut remains passively dilated (light-green) or passively occluded (dark-green). Red, orange and yellow areas represent active contractions and mark the propagating area of contraction during neural peristalsis. Active relaxation (cyan and light-blue) precedes both in time and space the propagating contraction. In **(c)** we present a simplification of the full composite map of states by clustering all areas undergoing active contraction (red) and, active relaxation (blue). All other passive states are in white. **(d)**–**(f)** show an example of an instant (vertical line in **(a)**–**(c)**; ∼ 3.5 minutes) where the liquid bolus between 190 mm and 230 mm is traveling down the length of the colon from left to right. The actual rabbit colon is shown in **(d)**. In **(e)** the gut is color coded with the colors from the multiple mechanical states shown in **(b)**. In **(f)** the simplified mechanical states from **(c)** are shown. The isotonic contraction at 190 mm (orange) and auxotonic contraction at 180 mm (yellow) push the bolus to the right, while the isometric contraction at 150–170 mm (red) ensures no back-flow. The isotonic relaxation at 210–240 mm (light-blue) help to expand the colon to accommodate the incoming bolus. The distended quiescence at 200 mm (light-green) depicts a momentary stillness where changes in diameter and pressure are trivial. The isometric relaxation between 80–140 mm (dark-blue) represents the recovery from earlier active contractions in that region that resulted in propelling the liquid bolus to the right.

The optimal state sequence for each of the sensor locations (0–250 mm at 10 mm intervals) was independently inferred. When visualising, each state sequence was placed according to the location of the corresponding sensor in the colon, resulting in a 2D map where horizontal sequence strips are vertically stacked, shown in Figure 6 (a)–(c).

Parameters of the hidden Markov model were chosen manually, giving robust results with respect to variations in the parameters, shown in Figure 5. The *γ* parameter specifying transition probabilities should be adjusted to account for variations in time sampling frequency, which was not required here as our data consisted entirely of 4 Hz sampling.

The states inferred by our method coincided with hypothesised states based on manual observation of orbital plots and the expected mechanical function of the colon. This allowed qualitative analysis of colon dynamics as given in the description of Figure 6(d)–(f). A more detailed description of these finding can be found in our previous paper [4].

## Conclusions

The use of the hidden Markov model to discriminate mechanical states of the intestinal muscle in an isolated preparation of the rabbit colon has given experimental neurogastroenterologists a novel powerful tool to identify the active and passive states of the intestinal muscle.

The graphic representation of where the active contractions and relaxations occur in the intestine at any particular time (Figure 6) will allow for the testing of many hypotheses currently proposed but not validated on the mechanisms responsible for the appropriate mixing and propulsive movements [4].

At the present the parameters described here have been shown to work with the rabbit distal colon. Whether or not the same criteria could be applied to different species with different sized diameters is still to be determined. It is likely that the parameter will need to be adjusted for each species (including human). We are currently setting up studies with different animal species to test this hypothesis.

## Endnotes

^a^ [P] = 1 if P is true, 0 if P is false.

^b^**1**_*A*_(*x*) = 1 if *x* ∈ *A*, and 0 otherwise.

## Competing interests

The authors declare that they have no competing interests.

## Authors’ contributions

LW, MC, and PD - study concept and design, drafting of the manuscript; critical revision of the manuscript for important intellectual content. PD - recorded the data. LW - software development and analysis. All authors read and approved the final manuscript.
